# Trends in Socioeconomic Inequalities in Breast Cancer Incidence Among Women in Canada

**DOI:** 10.1177/10732748231197580

**Published:** 2023-08-22

**Authors:** Madeline Tweel, Grace M. Johnston, Mohammad Hajizadeh

**Affiliations:** 1 12361 Faculty of Medicine, Dalhousie University, Halifax, Canada; 2 School of Health Administration, 3688 Dalhousie University, Halifax, Canada; 3 Beatrice Hunter Cancer Research Institute, Halifax, Canada

**Keywords:** breast neoplasms, health status disparities, socioeconomic factors, incidence, Canada

## Abstract

**Introduction:**

Breast cancer is the most common cancer among females in Canada. This study examines trends in socioeconomic inequalities in the incidence of breast cancer in Canada over time from 1992 to 2010.

**Methods:**

A census division level dataset was constructed using the Canadian Cancer Registry, Canadian Census of the Population and National Household Survey. A summary measure of the Concentration index (C), which captures inequality across socioeconomic groups, was used to measure income and education inequalities in breast cancer incidence over the 19-year period.

**Results:**

The crude breast cancer incidence increased in Canada between 1992 and 2010. Age-standardized C values indicated no income or education inequalities in breast cancer incidence in the years from 1992 to 2004. However, the incidence was significantly concentrated among females in high income and highly educated neighbourhoods almost half the time in the 6 most recent years (2005–2010). The trend analysis indicated an increase in breast cancer incidence among females living in high income and highly educated neighbourhoods.

**Conclusion:**

Breast cancer incidence in Canada was associated with increased socioeconomic status in some more recent years. Our study findings provide previously unavailable empirical evidence to inform discussions on socioeconomic inequalities in breast incidence.

## Introduction

Cancer is a major public health issue in Canada because it is a leading cause of death and morbidity. Breast cancer is the most diagnosed cancer among females with an estimated 28,900 cases in 2022.^1^ One in eight females are expected to develop breast cancer during their lifetime.^2^

While the aetiology of breast cancer is poorly understood, several risk factors have been identified. Like many cancers, older age is a risk factor, with the majority of new breast cancer cases occurring in females aged 50–80 years.^3,4^ Other risk factors for breast cancer include reproductive factors (i.e., young age at menarche, late age at menopause, late age at first pregnancy, and the low number of pregnancies), greater oestrogen exposure, ethnicity, and various lifestyle factors (smoking is a risk factor for breast cancer^4^ while greater physical activity and lower BMI^5,6^ are found to be protective factors).^7^ Lifestyle and oestrogen-related risk factors are potentially mutable.

Socioeconomic status (SES) is one of the main determinates of health.^8^ SES may affect cancer incidence through variation in exposure to risk factors and lifestyles.^9^ While higher incidence of some cancers appears to be associated with lower SES,^10,11^ higher breast cancer incidence is often associated with higher SES.^12-14^ Such findings have been reported in Europe,^15-19^ Asia,^19,20^ Brazil,^21^ New Zealand,^22^ Australia,^23^ and the United States.^19^ In Canada from 1992 to 2004, breast cancer incidence rates were greatest among neighbourhoods in the highest income quintile.^24^ While the latter study examines the relationship between SES and breast cancer incidence in Canada, it does not measure changes in the association between SES and breast cancer incidence over time. This paper addresses the information gap. Using a commonly used measure of SES inequality in health,^10,11^ we determined the trends in education and income inequalities in the incidence rate of breast cancer among Canadian females from 1992 and 2010. This is the period in which the Canadian Cancer Registry (CCR) data were available for all provinces at the Statistics Canada’s Research Data Centres. Understanding the relationship between breast cancer incidence and change in SES patterns over time can help address unacceptable inequalities in the occurrence of breast cancer.

## Methods

### Data and Variables

SES inequalities in breast cancer incidence in Canada were examined using Census Division (CD) level data (n = 280) constructed from the CCR, the Canadian Census of the Population (CCP, 1991, 1996, 2001, 2006), and the National Household Survey (NHS 2011). Using the International Classification of Diseases for Oncology (ICD-O-3) code C50, Canadian females with breast cancer were identified from the CCR. The Postal Code Conversion File Plus (PCCF+) Version D software was used to determine the CD coordinates for breast cancer cases based on postal code data available in the CCR. Then, the number of breast cancer cases was calculated for each CD.

The CCR is a registry of all primary cancer diagnoses collected from administrative data by each provincial/territorial cancer registry and reported to Statistics Canada. The CCR is one of the highest quality national population-based cancer registry systems in the world.^2^ The CCR does not have information on SES indicators; thus, the CCP and NHS were used to obtain CD-level SES variables. These indicators include CD-level average and median equivalized household income and the proportion of individuals with a bachelor’s degree and above. To account for household sizes, household incomes were equivalized by dividing them by the square root of household size^25^ when we calculated CD-level average and median household income. Using the total number of females residing in each CD obtained from the CCP and NHS and the total number of breast cancer cases derived from the CCR, we estimated breast cancer incidence for each CD. Breast cancer incidence for each CD was then linked to the SES indicators and age characteristics of females obtained from the CCP and NHS. Data linkage is detailed in Table 1. The constructed CD-level dataset was used to measure the crude incidence and age-standardized income and education inequalities in breast cancer incidence over the period studied.

**Table 1.** Source of Socioeconomic and Demographic Information Linked With the Canadian Cancer Registry Data.

Sources of socioeconomic and demographic information (year)	Years of the Cancer Registry linked
Canadian census of the population (1991)	1992 – 1993
Canadian census of the population (1996)	1994 – 1998
Canadian census of the population (2001)	1998 – 2003
Canadian census of the population (2006)	2004 – 2008
National household survey (2011)	2009 – 2010

### Statistical Analysis

#### Measuring Crude Incidence Rates

The estimated breast cancer incidence rates for CDs were used to calculate the crude incidence rate for Canada and its provinces. The population size of each CD was used as a weight in the calculation of crude incidence rates. The crude cancer incidence rates provide the real number of new cancer cases which can be used in designing primary and secondary prevention and treatment cancer strategies.

#### Measuring Income and Education Inequalities

Income and education inequalities in breast cancer in Canada were measured using the Concentration index (C). Other measures, including the index of dissimilarity and the Gini index, have been used in the literature to examine inequalities in health outcomes. However, the C is a preferred measure of socioeconomic inequality as it accounts for health inequalities arising from SES factors, it represents the entire population, and it is responsive to variations in the distribution of the population among different SES groups.^26^

The C reflects the distribution of health outcomes across all SES groups. The C is calculated using the Concentration curve, which is a plot of the cumulative percentage of the population ranked by SES indicators (e.g. income or education) on the *X*-axis and cumulative percentage of health outcome (breast cancer incidence) on the *y*-axis. The C is defined as twice the area between the Concentration curve and the line of perfect equity (45-degree line). The value of C ranges between +1 and −1, where zero indicates perfect equality, that is, no inequality. A negative value indicates the health outcome is higher among lower SES groups, while a positive value indicates the health outcome is more concentrated among higher SES groups.^27^

Age-standardized income (education) inequality can be calculated using an indirectly standardized C by including the age standardizing variables (16 age-group variables including 15 five-year age-group plus an open-ended age group 85+, except for a reference group) in the ‘convenient regression’ as^28^:

(1) $$2\sigma_{r}^{2}\left(\frac{{BCa}_{i}}{M}\right)=\alpha +\theta r_{i}+\overset{16}{\underset{k=1}{\sum }}{\beta_{k}age\_g}_{ki}+\varepsilon_{i},$$

where *BCa*_*i*_ denotes CD *i*’s breast cancer incidence rate, *M* is the mean incidence rate for breast cancer for all CDs, *α* is the intercept, and *r*_*i*_ is the CD *i*’s fractional rank in the SES distribution and is calculated as *r*_*i*_=*i*/*n* (*i*=1 for the CD with lowest SES and *n* for the CD with highest SES). The $\sigma_{r}^{2}$ is the variance of fractional rank. *age*_*g*_*ki*_ is the age group *k* from the total population of the CD *i* and *β*_*k*_ is the corresponding coefficients for *age*_*g*_*k*_. The ordinary least squares (OLS) estimate of *θ* and its standard error in Equation (1) give the magnitude and the standard error for the age-standardized C. In the calculation of age-adjusted C, the population size of each CD was used as weights.

#### Analyzing Trends in the Incidence and Income and Education Inequalities

Trend analyses were performed by plotting time (19 points corresponding to the years 1992–2010) against breast cancer incidence. The slope of the regression line was used to determine linear trends in crude breast cancer incidence over time. Additionally, trend analyses were performed by plotting time (19 points corresponding to the years 1992–2010) against the age-standardized C of each inequality measure. The slope of the regression line was used to determine the linear trend of the age-standardized C overtime at a *P*-value <.05.

## Results

### Crude Breast Cancer Incidence

Table 2 presents the crude incidence of breast cancer in females across the Canadian provinces from 1992 to 2010. The crude breast cancer incidence in Canada increased significantly from 1992 to 2010. The results show an increase of 1.27 cases/100,000 people per year from 115 cases/100,000 people in 1992 to 136 cases/100,000 people in 2010. The increased incidence was seen for all Canadian provinces, except for British Colombia. The increased incidence was greatest for the Atlantic provinces (New Brunswick, Newfoundland and Labrador, Nova Scotia, and Prince Edward Island) and Quebec. Newfoundland and Labrador saw the greatest increase at 2.96 cases/100,000 people per year.

**Table 2.** Crude Incidence of Breast Cancer in Females per 100,000 Across the Canadian Provinces From 1992-2010.

Year	Canada	Provinces									
		AB	BC	MB	NB	NL	NS	ON	PE	QC	SK
1992	115	104	134	118	104	84	112	116	116	112	124
1993	114	99	137	121	117	80	111	114	77	114	121
1994	109	93	114	123	113	83	124	108	133	113	116
1995	112	92	116	129	115	94	125	111	104	116	111
1996	113	102	118	126	119	94	120	112	111	117	109
1997	121	115	128	122	120	94	127	120	126	122	123
1998	125	108	129	125	127	96	140	121	163	135	120
1999	125	113	126	134	129	102	134	120	124	136	127
2000	124	111	120	126	124	110	139	117	132	141	118
2001	125	109	122	131	128	122	131	120	110	140	124
2002	129	122	127	131	129	110	146	127	124	139	127
2003	126	117	122	128	135	112	128	122	161	137	124
2004	123	110	118	130	121	115	132	120	144	134	125
2005	127	105	126	129	141	125	143	125	116	136	126
2006	129	110	124	131	136	117	153	126	108	140	129
2007	132	115	130	133	138	125	138	130	116	143	133
2008	133	113	133	136	138	138	150	128	152	145	130
2009	132	111	132	133	142	125	146	128	127	145	136
2010	136	118	138	132	141	133	155	134	176	145	129
Average 1992-2010	124	109	126	128	127	108	134	121	127	132	124
Trend coefficients (*P*-value)	1.27 (<.001)	.92 (.003)	.35 (.241)	.69 (<.001)	1.78 (<.001)	2.96 (<.001)	1.98 (<.001)	1.10 (<.001)	1.92 (.049)	1.92 (<.001)	.92 (<.001)

Note. BC: British Columbia; AB: Alberta; SK: Saskatchewan; MB: Manitoba; ON: Ontario; QC: Quebec; NB: New Brunswick; NS: Nova Scotia; PE: Prince Edward Island; NL: Newfoundland and Labrador.

### Income and Education Inequalities in Breast Cancer Incidence

Table 3 reports income and education inequalities in breast cancer incidence in Canada over the study period. Although most age-standardized C values for average household equivalized income were positive, significant age-standardized C values were only seen for years 2005 and 2010; both values were positive. This indicates breast cancer incidence was concentrated more among females in higher average income neighbourhoods in 2005 and 2010. Trend analysis indicates a significant increase in the incidence of breast cancer in higher average income neighbourhoods from 1992 to 2010.

**Table 3.** Age-Standardized Income and Education Inequalities in Breast Cancer Incidence in Canada From 1992 to 2010.

Year	Age-standardized C (95% confidence interval)		
	Average household equivalized income	Median household equivalized income	Education (Bachelor’s degree or higher)
1992	.001 (−.031 to .032)	.005 (−.026 to .037)	−.016 (−.082 to .05)
1993	−.01 (−.042 to .023)	−.001 (−.033 to .032)	−.038 (−.106 to .03)
1994	−.012 (−.032 to .008)	−.015 (−.033 to .004)	.011 (−.008 to .03)
1995	−.018 (−.04 to .003)	−.011 (−.03 to .009)	−.018 (−.039 to .003)
1996	−.008 (−.027 to .01)	−.007 (−.024 to .011)	.014 (−.012 to .04)
1997	.011 (−.01 to .032)	.011 (−.007 to .03)	.017 (−.006 to .04)
1998	−.01 (−.03 to .011)	−.002 (−.02 to .016)	−.003 (−.024 to .018)
1999	.002 (−.016 to .02)	.001 (−.016 to .018)	.005 (−.016 to .025)
2000	−.015 (−.037 to .006)	−.015 (−.034 to .004)	−.004 (−.028 to .021)
2001	.006 (−.012 to .025)	.004 (−.012 to .02)	.009 (−.01 to .028)
2002	.002 (−.018 to .022)	−.004 (−.023 to .015)	.011 (−.009 to .03)
2003	.006 (−.014 to .025)	.004 (−.013 to .022)	.012 (−.009 to .033)
2004	.013 (−.002 to .028)	.013 (−.002 to .028)	.006 (−.011 to .023)
2005	.014 (.001 to .028)*	.012 (−.001 to .025)	.021 (.005 to .038)*
2006	.009 (−.008 to .027)	.013 (−.004 to .031)	.018 (−.001 to .038)
2007	.014 (−.004 to .032)	.021 (.005 to .037)*	.023 (.008 to .038)*
2008	.00 (−.019 to .019)	.007 (−.013 to .026)	.02 (.003 to .037)*
2009	.011 (−.003 to .025)	.012 (−.001 to .025)	.024 (.007 to .041)
2010	.024 (.007 to .04)*	.022 (.005 to .038)*	.018 (−.002 to .039)*
Trend coefficients (*P*-value)	.002 (<.001)	.001 (<.000)	.002 (.003)

*P-value <.05.

**Figure 1.**
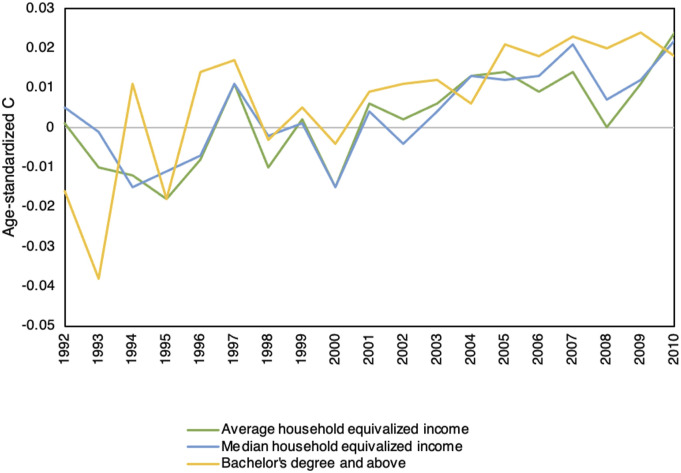
Trends in income and education inequalities in breast cancer incidence among females in Canada: 1992 to 2010.

Similarly, although age-standardized C values for median household equivalized income were positive in most years, the age-standardized C values were only significant for years 2007 and 2010. Both values were positive, indicating breast cancer incidence was higher among neighbourhoods of higher median income in 2007 and 2010. Trend analysis indicates a significant increase in the incidence of breast cancer among higher median income neighbourhoods from 1992 to 2010, as illustrated in Figure 1.

Age-standardized C values for education inequalities were positive in most years. Age-standardized C values were statistically significant for the years 2005, 2007, 2008, and 2010. The significant positive values of the age-standardized C indicated that breast cancer incidence was higher among high-educated neighbourhoods in these years. Trend analysis indicated a significant increase in the incidence of breast cancer among high-educated neighbourhoods from 1992 to 2010, as shown in Figure 1.

In summary, across the inequalities examined, age-standardized C values indicated no income or education inequalities in breast cancer incidence in the 13 years from 1992 to 2004. However, the incidence was significantly concentrated among females in high income and highly educated neighbourhoods in 4 of the 6 most recent years (2005–2010), and the trend analysis produced an increase in breast cancer incidence among females living in high income and highly educated neighbourhoods.

## Discussion

### Crude Breast Cancer Incidence

Our results found that crude breast cancer incidence increased in Canada between 1992 and 2010. This may be attributable to the fact that the likelihood of being diagnosed with breast cancer increases with age, and the Canadian population is ageing. The increasing crude incidence means that a higher percentage of women are being diagnosed with breast cancer over time, highlighting the urgency of examining patterns of its occurrence. We reported crude incidence rates, which are not as readily available as age-standardized incidence rates, to reflect what has actually happened.

From an examination of the incidence rates by province and their change over the study time period (Table 1), mammography screening practices might also have had an impact. When screening programs are introduced, incidence rates initially increase because cases are detected earlier than would have been the situation if the screening program had not been introduced.^29^ British Columbia was the first province to implement a formal breast screening mammography program in 1988 which is a few years before 1992, the initial year of our study dataset. This might explain why British Columbia was the province with the lowest increase in their mammography rates from 1990 to 2008,^29^ and had no clear decline in crude incidence from 1992 to 2010.

It took a decade, until 1998, for all provinces to have organized mammography screening, and before 2000/2001, mammography use varied substantially by province.^29^ Opportunistic screening was also occurring for women of all ages prior to and after screening programs were introduced.^29,30^ The increase in mammography use in the 1990s reached a peak in 2000/2001 followed by a stabilization in rates thereafter for women 50 to 69.^29^

### Income and Education Inequalities in Age Standardized Breast Cancer Incidence

Our age-standardized C values indicate breast cancer incidence was concentrated among high-income and more educated neighbourhoods in some years from 2005 to 2010. This finding is consistent with the literature which suggests that breast cancer incidence is associated with higher SES.^12-14^ Across Europe, Asia, and the United States women with higher education levels had increased breast cancer incidence.^19^ In New Zealand, breast cancer incidence was significantly higher in women with high income.^22^ In China, breast cancer incidence was higher in areas of greater socioeconomic development.^20^ In Brazil, breast cancer incidence was higher in regions with higher sociodemographic index (SDI, a measure of regional development based on income, educational attainment, and total fertility rate).^21^ In Australia, women of higher SES had a higher incidence of breast cancer.^23^ In Italy, increasing social level of the husbands’ occupation and increased number of years of schooling was associated with greater breast cancer risk.^18^ In Spain, an increased risk of breast cancer was associated with higher SES.^17^ In Norway, women with the highest level of education were found to have higher incidence of breast cancer,^16^ and increased education and income level was positively associated with incidence of localized and regional disease.^15^

Brinton and colleagues note that higher breast cancer incidence among women of higher SES may in part reflect trends in reproductive risk factors.^31^ Women of higher income and education have been shown to postpone childbearing and have fewer or no children,^32^ and this has been associated with a greater risk of breast cancer.^33^ Compared to women who have had three or more births, women who have not given birth have a risk of developing breast cancer that is twice as high. As well, women who first give birth over age 35 have a 2 to 3 times higher risk of developing breast cancer compared to women who give birth before age 20.^31^

Increased breast cancer risk has also been associated with exposure to exogenous hormones including oral contraceptives.^31^ One Canadian study indicates that lower annual household income was found to be associated with decreased use of oral contraceptives,^34^ and the most important barrier to accessing contraception was found to be cost.^35^ The Collaborative Group on Hormonal Factors in Breast Cancer found that those who were taking or had taken oral contraceptives within the last year had a 24% increased risk of having breast cancer. However, the risk decreased over time and posed no significantly greater risk after 10 years without an oral contraceptive.^36^

This leaves us to ask why we did not observe higher breast cancer incidence among higher SES women from 1992 to 2004. As complex relationships among risk factors for breast cancer are not well-understood, trends in breast cancer incidence are difficult to explain.^37^ Before the time period of our study data and the introduction of organized mammography screening across Canada, it was recognised that well-established primary risk factors for breast cancer incidence were associated with higher SES.^38,39^ Higher income and education attainment were also found to be associated with an increased breast cancer screening attendance.^40^ Thus, both primary risk factors and screening may contribute to the association between higher income and increased breast cancer incidence.^21^ However, the introduction and increase in mammography screening might be the reason why we did not observe the expected association between breast cancer incidence and high SES across the 1992 to 2004 time period in Canada.

In Canada, mammography use varied by income quintile and level of education. A Statistics Canada report of trends from 1990 to 2008, found that females 50 to 69 years with lower household income and education had lower rates of mammography use.^29^ However, that paper also reported that the gap between the middle- and higher-income women narrowed across the decade from 1990 to 2000. We speculate that this might be a reason why we did not observe significant socioeconomic inequalities from 1992 to 2004; this was the time period when the SES gap in mammography rates was narrowed by an increase in mammography by middle-income women. Subsequently though, from 2005 to 2008, the mammography rates of the lowest income quintile declined.^29^

### Study Strengths and Limitations

The strengths of this study are the unique linkage of large amounts of data from the CCR and SES data from the CPP and NHS and the use of a summary measure of the C, which helped us to quantify and examine trends in income and education inequalities in breast cancer incidence in Canada. There are, however, some limitations to this study. First, SES was measured using area-based indicators, which does not account for individual characteristics. Both area- and individual- and area-level SES were found to have independent associations with health outcomes;^41^ thus, future studies using both individual- and area-level SES can provide a more comprehensive picture of the relationship between SES and breast cancer incidence in Canada. Second, the CCP data are collected every 5 years, therefore the CCP data was linked to the CCR data of the closest year to estimate SES. We did not differentiate between types of breast cancer, and we did not include male sex in our study. Additionally, the CCR does not capture the ethnicity of individuals, limiting our ability to analyze race as a determinant of breast cancer incidence. Third, given the dataset at our disposal, we could not assess trends in socioeconomic inequalities in breast cancer staging at diagnosis. The existing studies suggest that most female breast cancer cases get diagnosed early in Canada^42^ and there are no socioeconomic status differences in the stage of diagnosis of breast cancer in Ontario, Canada.^43^ Fourth, given a cross-sectional design of our analysis to measure socioeconomic inequalities prevented us from establishing the temporal relationship between SES and breast cancer incidence analysis, thus impeding our ability to make causal inferences. Lastly, our study provided insight into income and education inequalities in the incidence of breast cancer in Canada over almost 2 decades; however, we could not investigate the association between SES and breast cancer incidence in recent years due to the data availability. The findings need to be updated using more recent data when they become available in the future.

## Conclusion

Breast cancer incidence appears to be more concentrated among females of high income and education neighbourhoods in some recent years. These patterns in incidence may be related to differences in reproductive and lifestyle factors among women of higher SES compared to lower SES. The introduction of mammography might have reduced the association between high income and higher incidence for more than a decade, from 1992 to 2004, before the historical pattern of higher incidence for higher SES women began to re-emerge (2005–2010). Our findings help to address an important empirical gap in the literature and propose a possible SES moderating effect during the time period when mammography was introduced. Further research is advised to extend and more fully understand trends in the relationship between SES and breast cancer incidence.
